# Epidemiology of Moyamoya Angiopathy in Eastern India

**DOI:** 10.3389/fneur.2022.837704

**Published:** 2022-03-04

**Authors:** Shambaditya Das, Souvik Dubey, Suman Das, Avijit Hazra, Alak Pandit, Ritwik Ghosh, Biman Kanti Ray

**Affiliations:** ^1^Department of Neurology, Institute of Post Graduate Medical Education & Research, Bangur Institute of Neurosciences, Kolkata, India; ^2^Department of Pharmacology, Institute of Post Graduate Medical Education & Research, Kolkata, India; ^3^Department of General Medicine, Burdwan Medical College and Hospital, Burdwan, India

**Keywords:** Moyamoya disease, epidemiology, Stroke, India, Moyamoya angiopathy, Moyamoya syndrome, childhood stroke

## Abstract

**Introduction:**

Moyamoya angiopathy (MMA) is a chronic, progressive intracranial vasculopathy with variation in prevalence and clinical manifestations across different populations. This study was aimed to estimate the frequency of MMA as an etiology of stroke and its epidemiological features in the largest cohort of MMA patients in India.

**Method:**

A single-centered cross-sectional observational study over a period of 5 years (2016–2021) was undertaken among consecutive stroke and transient ischemic attack (TIA) patients to look for the presence of MMA angiographically. Each patient with angiographically proven MMA was further evaluated for demographic, clinical, and radiological characteristics.

**Results:**

Among 10,250 consecutive stroke and TIA patients (ischemic = 78%, hemorrhagic = 22%), frequency of MMA was 1.56% (*n* = 160); 15.3% among children. Female preponderance (Male:Female = 1:1.4) was noted among 160 MMA patients, with bimodal age distribution, first peak at 3–8 years, and a shorter second peak at 41–47 years. Childhood-onset MMA was seen in 75 (46.9%) with commonest initial neurological symptom of fixed-motor-weakness (44.0%), followed by TIA (26.7%); while 85 (53.1%) had adult-onset MMA with fixed-motor-weakness (50.6%) followed by headache (24.7%) as the predominant initial neurological symptom; seizure significantly higher in children (*p* < 0.001) and headache in adults (*p* = 0.012). Transient and fixed neurological manifestations constituted 87.5 and 69.4% respectively, of symptoms throughout the disease course. Cerebral infarction (45.0%) and TIA (21.9%) were the commonest types of MMA. On brain imaging, infarction was noted in 80.6%, hemorrhage in 11.3%, significantly higher among adults (*p* < 0.001). Cortical infarct and Gyral pattern were commoner in children (*p* = 0.004), subcortical infarcts in adults (*p* = 0.018). Frequent Suzuki staging observed was stage 4 (31.3%), followed by stage 3 (30.0%). Involvement of posterior circulation was detected in 55.6%, brain atrophy at the time of diagnosis was seen in 65.0%.

**Conclusion:**

MMA is an important etiological consideration in patients with stroke, especially in children. It can present with a myriad of transient neurological symptoms, frequently overlooked, leading to delayed diagnosis, and contributing to socio-economic burden. Indian MMA showed aberrations in its gender predisposition, age distribution, frequency of familial cases, disease manifestation, and type of stroke, in comparison to its Japanese and Caucasian counterparts pointing to the inter- and intra-continent differences of MMA phenotype. Future development of the Indian MMA national registry is of essence.

## Introduction

Moyamoya angiopathy (MMA) is a chronic progressive occlusive intracranial vasculopathy characterized by steno-occlusive lesions of the terminal internal carotid artery (ICA) or proximal anterior cerebral artery (ACA) and/or middle cerebral artery (MCA) with the formation of an abnormal vascular network at the base of the brain, classically appearing as a “puff of smoke” (1). MMA can be divided into moyamoya syndrome (MMS) or quasi-moyamoya (those with a well-recognized associated condition and angiographic evidence of uni/bilateral stenosis) and moyamoya disease (MMD, those without any associated disorder and bilateral stenosis) (2, 3).

The etiology of MMA is yet to be fully elucidated. However, recent genetic studies have identified RNF213 as an important susceptibility gene of MMA among East Asian populations (4–6). Possibly due to these genetic differences, the frequency and epidemiological features of MMA between East Asian countries like Korea and Japan, vary from those in the Western Hemisphere. Besides, differences have been observed in prevalence and MMA phenotype between countries within Asia (4–21). Due to these regional variations, extrapolation of knowledge from Japanese MMA to the Indian population may be unwise (22). However, only limited data exist pertaining to MMA in the Indian population, with no nation-wide epidemiological study till date (14, 23–31). Nation-wide studies on MMA epidemiology are available for only a few countries like Japan, Korea, Taiwan, China, and the USA (4, 9–11, 17, 20, 21). MMA though uncommon, the disease burden is still heavy due to the high hospitalization rate, long hospital stays, and increasing costs. Besides, delayed diagnosis is associated with greater mortality and morbidity putting significant socio-economic strain on public health systems and their families (10, 14, 17). The development of a national database for a relatively rare disease like MMA in India, a lower-middle-income country (32) with limited financial capacity and insufficient health resources, may not be easy. Our institute is the largest tertiary care referral center in Eastern India catering to a large population with wide geographical representation and thus may be representative of the MMA status of a significant proportion of India. This single-center study was undertaken to estimate the frequency of MMA as an etiology of stroke and its epidemiological features in the largest cohort of MMA patients in India. These data could provide important clues to understand the pathogenesis, disease phenotype, and its socio-economic implications and inform policy-makers for the management of MMA.

## Materials and Methods

A descriptive, observational, and cross-sectional study was undertaken from a single, tertiary-care-center over a period of 5 years (2016–2021), screening consecutive stroke and transient ischemic attack (TIA) patients with intracranial angiography (non-invasive or invasive angiography, decided on individual case basis) to look for moyamoya angiopathic changes. All patients with evidence of MMA on angiography were recruited in the study.

### Demographic, Clinical, and Radiological Analysis

Every patient was thoroughly evaluated by history taking, physical examination, and investigations in order to assess the symptomatology, elicit clues to any associated underlying condition and radiographic features. The following epidemiological characteristics were included: age at the time of onset of neurological symptom (>12 years taken as adult and up to 12 years taken as child), gender, area of residence (rural/urban), and family history of similar illness. The clinical characteristics documented were age at the time of diagnosis, initial symptomatology at the onset, symptom leading to diagnosis, recurrence, any immediate precipitating, and associated clinical condition (if any). Appropriate brain imaging was undertaken in form of CT scan, MRI, MR angiography (MRA), and digital subtraction angiography (DSA). CT scan (Philips, 16 slices) was the preferred initial investigation among patients with suspected hemorrhagic stroke. MRI and MRA [Siemens 3Tesla MRI machine (MagnetomVerio DOT, 16 channels) using a standard quadrature head coil] were done for every patient. DSA was performed in 32 of our patients. The images were read and interpreted by independent neuro-radiologists. The type of parenchymal lesion (infarct/hemorrhage ± intraventricular extension), area of insult, “ivy” sign, involvement of posterior circulation, brain atrophy, and Suzuki staging were documented from the radiological investigations.

Other investigations performed: Each patient was subjected to blood investigations that included complete blood count, erythrocyte sedimentation rate (ESR), serum electrolytes, blood sugar, lipid profile, high sensitivity C-reactive protein (hsCRP), coagulation profile, anti-nuclear antibody (ANA), vasculitis profile, thyroid profile, viral markers, screening for a hyper-coagulable state (serum homocysteine, protein-C, protein-S, anti-thrombin III, factor V mutation, and anti-phospholipid antibodies profile), hemoglobin electrophoresis, serum lactate, and serum angiotensin converting enzyme (ACE) levels. Basic cardiothoracic work-up (chest X-ray, electrocardiogram, and echocardiography) was done. Carotid Doppler was done for each patient. Additional work-up was done depending on the clinical need case-to-case basis.

### Statistical Analysis

SPSS 25 was used for statistical analysis. Data were summarized by routine descriptive statistics, namely mean and SD for numerical variables that are normally distributed, median and inter-quartile range (IQR) for skewed numerical variables, and counts and percentages for categorical variables. Numerical variables were compared between two groups by Student's independent samples *t*-test if normally distributed, or by Mann–Whitney *U*-test, if otherwise. For multiple group comparison of skewed variables, Kruskal–Wallis ANOVA was used followed by Dunn's test for *post-hoc* comparisons between two individual groups. Fischer's exact test or Pearson's chi-square test were employed for intergroup comparisons of categorical variables. Analyses were two-tailed and the statistical significance level was set at *p* < 0.05 for all comparisons. Linear correlations between numerical variables were explored by scatter plots and estimation of Spearman's rank correlation coefficient Rho with 95% CI.

## Results

Among the 10,250 consecutive patients of stroke and TIA screened over the 5 years, 9,760 (95.2%) were adults and 490 (4.8%) were children. The male:female (M:F) ratio among adults and children was 1.17:1 and 1.22:1, respectively. Among adults, 7,574 (77.6%) patients had ischemic stroke or TIA and 2,186 (22.4%) had hemorrhage. Childhood stroke was ischemic in 394 (80.4%) cases and hemorrhagic in 96 cases (19.6%). Among the 10,250 screened patients, 160 patients (1.56%) had evidence of MMA angiographically. Among them, 85 (53.1%) had adult-onset MMA and 75 (46.9%) had childhood-onset MMA. Adult-onset MMA comprised 0.87% of adult stroke and TIA etiologies, while 15.3% of the etiological fraction of childhood stroke and TIA was shared by MMA.

**Figure 1:**
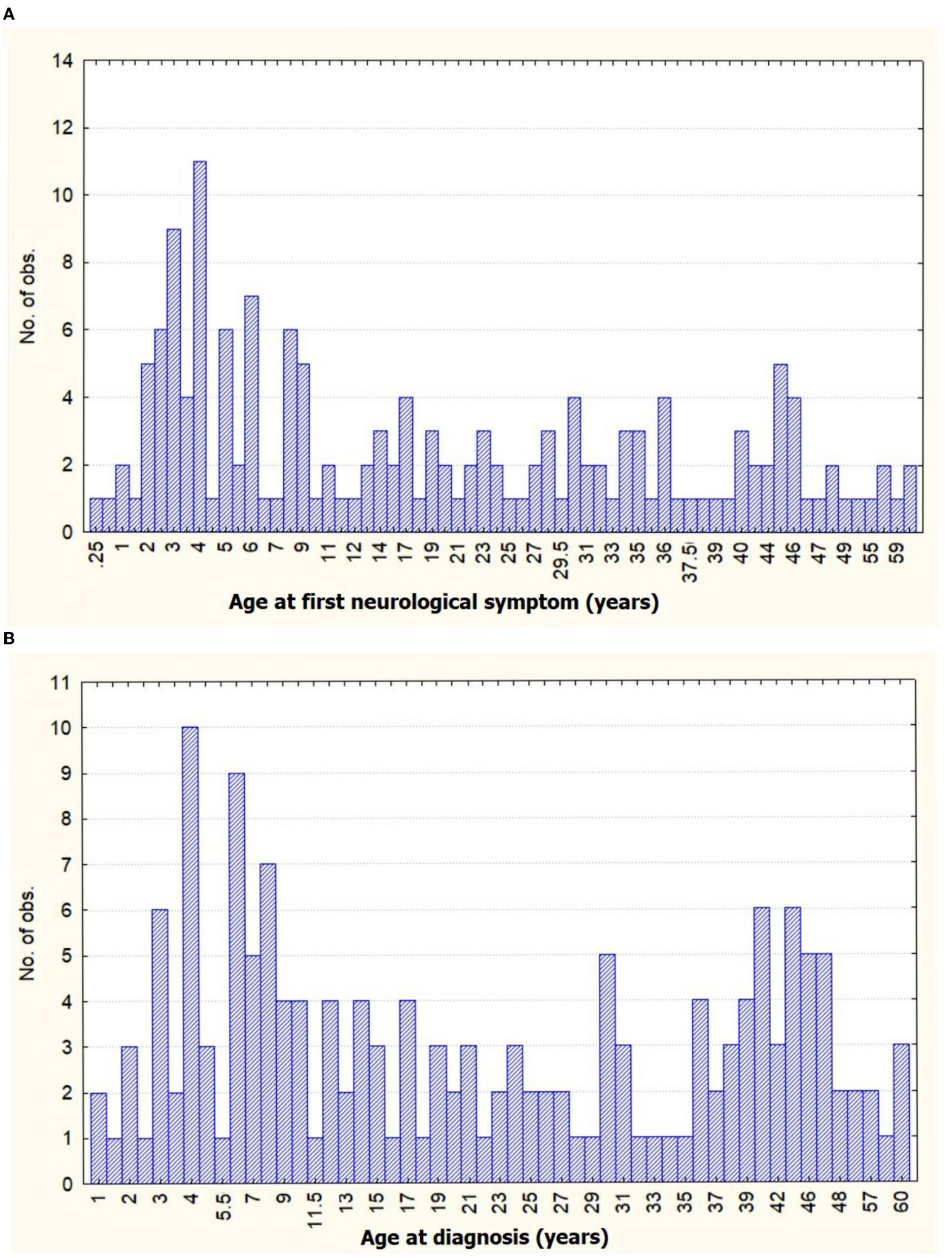
Distribution graph of age frequency with **(A)** age at first neurological symptom and **(B)** age of diagnosis of moyamoya angiopathy (MMA) in our cohort.

Among the 160 patients of MMA, a female preponderance was noted (M:F = 1:1.4), the female predominance was more marked among adults (M:F = 1:1.5). A majority (70.0%) of MMA patients were from rural areas. A family history of MMA was seen in two patients among 160 patients. The mean age of onset of first neurological symptoms for children was 5.1 ± 3.09 years (range 3.0–6.5 years), followed by a mean interval of 39.3 ± 60.37 months (median 18.0 months, range 0.0 to 42.0 months) and ultimately final diagnosis of MMA occurred at the mean age of 8.3 ± 6.52 years (range 4.0–11.0 years). For adults, the mean age of onset of first neurological symptoms was 33.7 ± 12.40 years (range 23.0–44.0 years), followed by a mean time gap of 22.5 ± 55.26 months (median 3.0 month, range 0.0–18.0 months), and diagnosed as MMA at the mean age of 33.5 ± 12.41 years (range 26.0–45.0 years). Thus, there was a statistically significant difference between child and adult in diagnostic latency (*p* = 0.001). A bimodal peak was observed in our cohort with the first peak between 3 and 8 years and a shorter second between 41 and 47 years (Figure 1). The history of precipitating factors (fever, heavy exercise, diarrhea, intake of hot spicy foods, taking a hot bath or cold water, crying, and emotional stress) was present among 41.3% of patients, statistically more in children (52.0%) than adults (31.8%). History of fever was predominant among all the precipitants (18.8%). Twelve out of 75 (11.11%) pediatric MMA cases were MMS (8 thalassemias, 1 neurofibromatosis-1, and 3 hereditary thrombophilia), while 24 out of 85 adults (25%) MMA cases were MMS (11 atherosclerotic, 3 thalassemias, 3 anti-phospholipid antibody syndrome, 1 systemic lupus erythematosus, 1 tubercular meningitis, and 2 Polycythemia) (Table 1).

**Table 1:** Clinico-demographic analysis of Moyamoya angiopathy (MMA) patients.

**Parameter**	**Overall**	**Children**	**Adults**	***P*-value**
	**(*n* = 160)**	**(*n* = 75)**	**(*n* = 85)**	
Sex ratio (M:F)	1:1.4	1:1.2	1:1.5	0.525
Area of residence Rural	70.0%	72.0%	68.2%	
Urban	30.0%	28.0%	31.8%	
Family history of MMA	2 (1.25)	1 (1.33)	1 (1.18)	1.000
Mean age at onset of first neurological symptoms (years)	20.3 ± 17.04 Median with range: 16.0 (4.3 – 35.0)	5.1 ± 3.09 Median with range: 4.0 (3.0 – 6.5)	33.7 ± 12.40 Median with range: 34.0 (23.0 – 44.0)	<0.001
Mean age at diagnosis (years)	22.8 ± 16.93 Median with range: 19.0 (7.0 – 38.0)	8.3 ± 6.52 Median with range: 6.0 (4.0 – 11.0)	33.5 ± 12.41 Median with range: 37.0 (26.0 – 45.0)	<0.001
Latency: first appearance of neurological symptoms to confirmed diagnosis (months)	30.4 ± 58.14 Median with range: 10.5 (0 – 24.0)	39.3 ± 60.37 Median with range: 18 (0 – 42.0)	22.5 ± 55.26 Median with range: 3 (0 – 18.0)	0.001
Presence of precipitating factor	66 (41.3%)	39 (52.0%)	27 (31.8%)	0.009
MMS: MMD	36: 124	12: 63	24: 61	0.087

Among children, the predominant first neurological symptom was fixed motor weakness (FMW) (44.0%), followed by TIA (26.7%), seizures (17.3%), headache (9.3%), visual symptoms (1.3%), and cognitive decline (1.3%). On the other hand, FMW was predominant (50.6%) first neurological complaint, followed by headache (24.7%), TIA (18.8%), seizure (2.4%), visual symptoms (2.4%), and cognitive and behavioral changes (1.2%) among adults. Seizure as both first (*p* < 0.001) and presenting symptom (*p* = 0.026) at the time of diagnosis was statistically significantly more prevalent among children than adults, while headache (*p* = 0.012) as the first symptom and TIA (*p* = 0.015) as presenting symptom were significantly higher among adults compared to children (Table 2). The transient and fixed neurological symptoms constituted 49.4 and 50.6%, respectively, as the initial symptom, 27.5 and 72.5%, respectively, at the time of presentation. Assessment of neurological symptoms throughout the disease course revealed perfusion-dependent transient neurological symptoms (*p* = 0.005) were significantly higher among the adults compared to children, while perfusion-independent transient neurological symptoms (*p* < 0.001) were significantly higher among the children compared to adult (Table 3).

**Table 2:** Symptomatology of MMA patients at different instances of disease course.

	**Symptomatology**	**Overall**	**Children**	**Adults**	***P*-value**
		**(*n* = 160) (%)**	**(*n* = 75) (%)**	**(*n* = 85) (%)**	
First neurological symptom	Fixed motor weakness	76 (47.5)	33 (44.0)	43 (50.6)	0.431
	Transient ischemic attacks	36 (22.5)	20 (26.7)	16 (18.8)	0.184
	Headache	28 (17.5)	7 (9.3)	21 (24.7)	0.012
	Seizures	15 (9.4)	13 (17.3)	2 (2.4)	<0.001
	Cognitive and behavioral changes	2 (1.3)	1 (1.3)	1 (1.2)	1.000
	Visual symptoms	3 (1.9)	1 (1.3)	2 (2.4)	1.000
	Extra-pyramidal symptoms	—	—	—	
Symptom at the time of presentation	Fixed motor weakness	103 (64.4)	42 (56.0)	61 (71.8)	0.047
	Transient ischemic attacks	19 (11.9)	14 (18.7)	5 (5.9)	0.015
	Headache	14 (8.8)	5 (6.7)	9 (10.6)	0.416
	Seizures	11 (6.9)	8 (10.7)	4 (4.7)	0.026
	Cognitive and behavioral changes	6 (3.6)	1 (1.3)	4 (4.7)	0.623
	Visual symptoms	5 (3.1)	3 (4.0)	2 (2.4)	0.666
	Extra-pyramidal symptoms	2 (1.3)	2 (2.7)	—	0.218

**Table 3:** Frequency of transient and fixed neurological symptoms at different instances of disease course.

	**Overall**	**Children**	**Adults**	***P*-value**
	**(*n* = 160)**	**(*n* = 75)**	**(*n* = 85)**	
	**(%)**	**(%)**	**(%)**	
**First neurological symptom**				
1. Transient neurological symptoms	79 (49.4)	40 (53.3)	39 (45.9)	0.267
(a) Perfusion dependent	64 (40.0)	27 (36.0)	37 (43.5)	0.423
(b) Perfusion independent	15 (9.4)	13 (17.3)	2 (2.4)	<0.001
2. Fixed neurological symptoms	81 (50.6)	35 (46.7)	46 (54.1)	0.428
**Symptoms at the time of presentation**				
1. Transient neurological symptoms	44 (27.5)	27 (36.0)	17 (20.0)	0.018
(a) Perfusion dependent	33 (20.6)	19 (25.3)	14 (16.5)	0.177
(b) Perfusion independent	11 (6.9)	8 (10.7)	3 (3.5)	0.026
2. Fixed neurological symptoms	116 (72.5)	48 (64.0)	68 (80.0)	0.080
**Symptoms during the course of illness**				
1. Transient neurological symptoms	140 (87.5)	68 (90.7)	72 (84.7)	0.339
(a) Perfusion dependent	104 (65.0)	40 (50.3)	64 (75.3)	0.005
(b) Perfusion independent	36 (22.5)	28 (37.3)	8 (9.4)	<0.001
2. Fixed neurological symptoms	111 (69.4)	50 (66.7)	61 (71.8)	0.497

The mean latency to reach the final diagnosis was greater if the first neurological symptom was transient (37.9 ± 67.87, range: 6.0–24.0), more pronounced if perfusion independent (84.8 ± 117.10, range: 9.0–141.0) compared to fixed neurological symptoms (23.0 ± 46.0, range: 9.0–141.0) (Table 4). Cerebral infarction (45.0%) was the most common type of MMA presentation, followed by TIA (21.9%); epileptic type of MMA was significantly higher among children (*p* < 0.001) and hemorrhagic presentation was significantly higher among adults (*p* < 0.001) (Table 5).

**Table 4:** Comparison between first neurological symptom and latency of diagnosis.

**First neurological symptoms**		**Overall**	**Children**	**Adults**	***P*-value**
		**(*n* = 160)**	**(*n* = 75)**	**(*n* = 85)**	
Transient neurological symptoms	All (Overall *n* = 79; child = 40)	37.9 ± 67.87 12.0 (6.0 – 24.0)	43.6 ± 67.33 24.0 (12.0 – 36.0)	31.6 ± 68.79 12.0 (0 – 24.0)	0.045
	Perfusion dependent (Overall *n* = 64; child = 27)	25.9 ± 42.02 12.0 (6.0 – 24.0)	29.4 ± 41.65 18.0 (6.0 – 36.0)	23.4 ± 42.71 12.0 (1.0 – 24.0)	0.125
	Perfusion independent (Overall n = 15; child = 13)	84.8 ± 117.10 24.0 (9.0 – 141.0)	71.1 ± 96.14 24.0 (12.0 – 120.0)	180.0 ± 254.56 180.0 (0 – 360.0)	0.937
Fixed neurological symptoms (Overall n = 81; child = 35)		23.0 ± 46.00 0 (0 – 24.0)	33.9 ± 51.22 0 (0 – 48.0)	15.1 ± 40.56 0 (0 – 7.0)	0.133

**Table 5:** Frequency of type of MMA presentation.

**Type of MMA**	**Overall**	**Children**	**Adults**	***P*-value**
**presentation**	**(*n* = 160)**	**(*n* = 75)**	**(*n* = 85)**	
TIA	35 (21.9)	20 (26.7)	15 (17.7)	0.184
Infarction	72 (45.0)	34 (45.3)	38 (44.7)	1.000
Hemorrhage	15 (9.4)	—	15 (17.7)	<0.001
Headache	21 (13.1)	7 (9.3)	14 (16.5)	0.242
Epilepsy	15 (9.4)	14 (18.7)	1 (1.2)	<0.001
Asymptomatic				
Others	2 (1.3)	—	2 (2.4)	0.499

Brain imaging revealed that infarcts were more common among children (88.0%) than adults (74.1%), while hemorrhage was seen predominantly among adults (20.0%) (*p* < 0.001). Considering the pattern of infarct, cortical infarcts and gyral patterns were significantly higher among children (*p* = 0.004), while the subcortical pattern was significantly higher among adults (*p* = 0.018). Posterior circulation stroke was seen in 6.3% of the patients. Brain atrophy was seen in 65.0% of our patients at the time of presentation. The “Ivy” sign was observed in 71.9% of patients and was observed significantly higher among children (84%) compared to adults (61.9%) (*p* = 0.002). Overall posterior circulation involvement was seen in 55.6% of cases. Most of the MMA cases fell under Suzuki stage 4 (31.3%), followed by stage 3 (30.0%) and stage 5 (20.6%) (Table 6). Among the radiological features, brain atrophy was statistically significantly associated with diagnostic latency (*p* < 0.001). A positive correlation was found between the gap and Suzuki staging with Spearman's coefficient of rank correlation (rho) 0.242 with significance level: *p* = 0.024 (Table 7).

**Table 6:** Radiological characteristics of MMA patients.

**Imaging parameter**		**Overall**	**Children**	**Adults**	***P*-value**
		**(*n* = 160) (%)**	**(*n* = 75) (%)**	**(*n* = 85) (%)**	
Type of lesion	Infarct	129 (80.6)	66 (88.0)	63 (74.1)	<0.001
	Hemorrhage	18 (11.3)	1 (1.3)	17 (20.0)	
	No acute insult	13 (8.1)	8 (10.7)	5 (5.9)	
Pattern of infarct	(A) Cortical	72 (45.0)	43 (57.3)	40 (47.1)	0.004
	- Gyral pattern	42 (26.3)	28 (37.3)	14 (16.5)	0.004
	(B) Subcortical	17 (10.6)	3 (4.0)	14 (16.5)	0.018
	(C) Both cortical and subcortical	33 (20.6)	17 (22.7)	16 (18.8)	0.564
	(D) Watershed	64 (40.0)	24 (32.0)	40 (47.1)	0.055
Posterior circulation stroke		10 (6.3)	7 (9.3)	3 (3.5)	0.191
Pattern of Hemorrhage	Intraparenchymal without IVH	4 (2.5)	1 (1.3)	3 (3.5)	0.002
	Primary IVH	6 (3.8)	-	6 (7.1)	
	Secondary IVH	8 (5.0)	-	8 (9.4)	
Brain atrophy		104 (65.0)	54 (72.0)	50 (58.8)	0.098
“Ivy” sign		115 (71.9)	63 (84.0)	52 (61.9)	0.002
Posterior circulation involvement		89 (55.6)	44 (58.7)	45 (52.9)	0.525
Suzuki staging	Stage 1	1 (0.6)	1 (1.3)	—	0.018
	Stage 2	26 (16.3)	6 (8.0)	20 (23.5)	
	Stage 3	48 (30.0)	25 (33.3)	23 (27.1)	
	Stage 4	50 (31.3)	30 (40.0)	20 (23.5)	
	Stage 5	33 (20.6)	12 (16.0)	21 (24.7)	
	Stage 6	1 (0.6)	1 (1.3)	—	

**Table 7:** Comparison of diagnostic latency with radiological features.

**Radiological**	**Mean ±SD**	**Median (IQR)**	***P*-value**
**feature**	**(months)**	**(months)**	
Brain Atrophy	37.8 ± 61.36	12.0 (0 – 36.0)	<0.001
“Ivy” sign	30.9 ± 55.44	12.0 (0 – 24.0)	0.267
Posterior circulation involvement	34.3 ± 61.02	12.0 (0 – 36.0)	0.106

## Discussion

Recent trends of MMA suggest an increasing number of cases from the entire globe. While apparently it may seem to reflect a growth in disease prevalence, a more plausible explanation seems to be increased diagnostic rates as a result of advances and increased accessibility to the non-invasive neuro-imaging techniques along with increased awareness among the neurologists regarding MMA (4, 14, 17). The incidence and prevalence of MMA are much higher in the Eastern Asian countries compared to their Western counterparts (4, 10, 12, 14, 17, 18, 33, 34). These differences can be majorly attributed to the distinct genetic background among the Asian population. A p.R4810K mutation in the ring finger protein 213 (*RNF213*), considered to be strongly associated with MMA, was detected in higher frequency among the Asians in comparison to the Caucasians. Additionally, differences in environmental and socio-economic factors might also contribute to this skewed distribution (10, 17, 35). The salient epidemiological characteristics of eastern and western hemisphere countries have been summarized in Table 8 (4–13, 15–21, 33, 35, 36). Among the Asian countries, Taiwan and China did not have the high incidence and prevalence of MMA seen among the Japanese and Korean populations. Besides, several studies have further pointed to ethnic and regional differences in the frequency of MMA within the same country. This is especially true for larger countries sheltering a heterogeneous population of dissimilar ethnicity. These observations may be attributed to the regional differences in effect size of p.R4810K mutation (4–6, 11, 16, 17, 20, 21). However, a direct comparison of these epidemiological data should be made cautiously primarily due to differences in the method of data collection, healthcare system, and population structure. While Japan and Korea have a wide availability of appropriate diagnostic tools and increased awareness among their primary treating physicians, the scenario in other countries of Asia is contrasting. Besides, Japan has a system of nationwide routine brain check-up system (the Brain Dock system) in place, which greatly enhances the chances of diagnosing asymptomatic MMA. Thus, the underestimation of asymptomatic MMA in other less economically developed countries in Asia is an actual possibility, owing to the less financial capacities among a larger population and a limited number of affordable healthcare facilities (4, 14, 17, 37).

**Table 8:** Salient epidemiological features of MMA based on recent data from countries around the world.

	**Incidence (per 100,000 person)**	**Prevalence (per 100,000 population-years)**	**Gender distribution (Female:Male)**	**Age distribution (years)**	**Familial occurrence (%)**	**Type of presentation (%)**	
						**Ischemic**	**Hemorrhagic**
**1. Eastern hemisphere countries**							
(i) Japan	0.54–0.94	6.03–10.5	1.8–2.2:1	Three peaks in male: 10–14, 35–39, 55–59, and two peaks in female: 20–24, 50–54.	10–15	48.5–66	19–21 (Adult: 32.5–51)
(ii) Korea	1.0–2.3	6.3–16.3	1.8–1.9	Bimodal: 5–14 and 45–54	10–15	38.3–49.6	42.4 (Adult: 62.4–69)
(iii) Taiwan	0.15–1.74	1.61	1.4:1	Bimodal: 5–9 (males), 10–14 (female), and 40–44	-	46.7	26.5
(iv) China	0.42– 0.59	0.72– 1.01	1.1:1	Bimodal: 5–9 and 35–45	5.2–7.5	40–70.8	14–55.9
**2. Western hemisphere countries**							
(i) Europe	0.03–0.07	0.8	1.8–3.2	Bimodal: 11–18 and 40–49	5.7	47–82	8.5–23
(ii) USA	0.086–0.57	–	1.4–4.3:1	Bimodal: 1st and 4th decade	2	21.4–79	6.4–29

Moyamoya angiopathy constituted 0.87% and 15.3% stroke etiology in adults and pediatric patients in our center's experience over the 5 years. The exact data on etiological distribution in adult stroke patients are limited and comparisons cannot be made. The frequency of MMA among pediatric stroke patients in our center was consistent with previous data from India (11.8–20%) (38–41). However, it was much less when compared to the recent studies in other Asian countries like Japan (30%), Korea (28.1%), and China (40.4–55.1%) (34, 40, 42–45). The International Pediatric Stroke Study Group (IPSSG), which included pediatric stroke patients of diverse ethnicity and geographic location, reported 6.5–11.6% of pediatric stroke as MMA (46, 47). Thus, discrepancies were likely related to genetic variation among ethnicities. A need for a national registry for MMA in our country is imminent. However, India is a large country with a heterogeneous population of diverse cultures and ethnicity coupled with the health and socio-economic burden of commoner infective diseases, the development of a national registry for rare diseases is challenging to say the least. The previous epidemiological observations on Indian MMA have been summarized in Table 9 (14, 23–25, 28–31).

**Table 9:** Representative epidemiological data on Indian MMA from previous literature.

	**Number (Female: Male)**	**Number of childhood MMA**	**Number of MMS included**	**Family history**	**Mean age of presentation (years)**	**Type of presentation (%)**		**Posterior circulation involvement (%)**	**Most common Suzuki staging (frequency)**
						**Ischemic**	**Hemorrhagic**		
**1. Northern India**									
(i) Garg et al. (28)	44 (1:2.1)	18 (41.9%)	0	N/A	25.6	31.8	68.2	N/A	N/A
(ii) Gupta et al. (31)	82	64 (78.1%)	0	N/A	14	81.7	8.5	N/A	Stage 5 (46.6%)
**2. Western India**									
(i) Tripathi et al. (30)	8 (1.8:1)	8 (100%)	0	N/A	7.6	100	0	12.5	N/A
(ii) Patil et al. (23)	41 (1:1.2)	41 (100%)	8 (19.5%)	Nil	6.3	92.7	0	N/A	N/A
**3. Southern India**									
(i) Sundaram et al. (25)	36 (1.3:1)	15 (41.7%)	10 (27.8%)	Nil	17.5	63.9	27.8	0	Stage 4 (47.2%)
(ii) Sadashiva et al. (24)	70 (1.1:1)	54 (77.1%)	0	N/A	13.8 (child- 8, adult- 33)	90	10	N/A	Stage 3 (76%)
**4. Eastern India**									
(i) Lahoti and Ray (29)	30 (1.2:1)	30 (100%)	0	Nil	6.7	96.7	3.3	13.3	Stage 3 (44%)
(ii) Das et al. (14)	76 (1.5:1)	36 (47.4%)	14 (18.4%)	Nil	18.1 (child-4.2, adult-31.5)	81.6	13.2	56.6	Stage 4 (41.7%)

Recent epidemiological studies have upheld the previous observation of relative female predisposition in MMA. While female predilection occurred at a frequency of 1.8–2.2 times in Japan and Korea, it was observed to be 1.8–4.25 times among the Western population (4, 10, 17, 36). In the present study, we observed a male-to-female ratio of 1:1.4. This attenuated female propensity was similar to other studies from Taiwan and China with a ratio of 1:1.1–1.4 and some of the other Indian studies (11, 16, 17, 24, 25). Although some Indian studies observed male predominance, caution must be exercised for interpretation because of possible bias from gender discrepancy in healthcare-seeking behavior in pre-select neurosurgical studies and relatively smaller study-population (23, 28, 48).

A relatively lower rate of familial occurrence of 1.3% was observed in our study, in comparison to recent Japanese and Korean (10–15%) and European (5.7%) studies. A similarly low rate of familial MMA was observed in the USA (2%). This further strengthens the idea of a strong genetic role in the epidemiological discrepancies in MMA. However, detection of the familial case greatly relies on the extensiveness of diagnostic work-up. It is suggested that asymptomatic immediate family members of MMA patients should be subjected to screening with transcranial Doppler (TCD) study followed by MRA, given the likelihood of MMA is 30–40 times higher among first-degree relatives in comparison to the general population (4, 7, 14, 17). In our study, we screened only those families of moyamoya patients who had neurological complaints consistent with stroke or TIA. A lack of uniform screening of asymptomatic family members of MMA patients in our cohort might have led to an underestimation of familial cases in our cohort. Future studies from India, using a sensitive diagnostic tool for familial screening, are warranted before drawing conclusions.

A bimodal age distribution with different clinical presentations is characteristically seen in MMA, with peaks occurring in the childhood and middle-aged group (4). A similar observation was made in our cohort. Though, the later peak among adults was comparable with Asian and Western countries (4, 10, 13, 15, 36), interestingly enough, the childhood peak commenced much earlier in our cohort. Deriving from the observation of increased incidence of fever (28%) prior to the onset of neurological symptoms among the children with MMA, significantly higher than adults (*p* = 0.008), a tenable explanation could be the “double hits” hypothesis, which states that systemic inflammation can potentiate onset and progression of MMA, in genetically susceptible individuals (49–52). Thus, a complex interplay of genetic and environmental factors might be the underlying mechanism behind the early onset occlusive vasculopathy among the children with MMA in our cohort. The recent epidemiological studies from around the globe have hinted toward a trend of the shift of the highest peak of incidence from children to adults, probably due to increased prevalence of comorbid stroke in adults and reclassification of previously misdiagnosed MMA for atherosclerotic disease due to improved diagnostic tools availability lately among adults (4, 7, 10, 17). However, for reasons unknown, childhood MMA peak remains predominant in our population. Close observation for future trends in our population is needed.

Moyamoya angiopathy can have transient or fixed neurological symptoms. The transient neurological manifestations are paroxysmal in nature with self-resolution and can further be categorized as perfusion dependent and perfusion independent. The perfusion-dependent transient neurological symptoms include TIA (paresthesia, motor-weakness, limb-shaking, amaurosis fugax, and dizziness), vascular type headache, and transient EPS like chorea. These are usually precipitated by transient decompensation of already compromised cerebral perfusion in MMA and are often seen in close temporal relation to maneuvers accentuating cerebral hypoperfusion (53–55). We observed a substantial number of patients (41.3%) with a history of an immediate precipitating factor in close temporal association to the onset of symptoms. The perfusion independent transient symptoms are sequelae to ischemic damage to the cerebral cortex, manifesting commonly as focal seizures. The fixed neurological symptoms occur as a result of a more permanent insult to the brain either due to ischemic or hemorrhagic events. The symptoms encompass FMW, cognitive and behavioral changes, higher-order visual dysfunction, and rarely fixed extra-pyramidal symptoms (14).

It is interesting to note that fixed neurological manifestations predominated (71.3%) the symptom leading to the diagnosis of MMA in our cohort. The first neurological symptom to appear was transient in nature in nearly half of our cases. Thus, it may be inferred that the diagnosis of MMA occurred majorly following the occurrence of a fixed neurological symptom. The initial subtle and transient symptoms were either missed or conveniently ignored by a first-in-contact physician and patient's kin, leading to an increased time gap between the onset of the first neurological symptom and eventual diagnosis of MMA in our patients (14). Diagnostic latency was significantly higher among children compared to adult MMA, and when the initial presentation was transient neurological symptoms compared to fixed symptoms (*p* = 0.001). It was highest when the first neurological symptom was a seizure, followed by a headache. Often, symptomatic mitigation of seizure and headache with anti-epileptics and pain-killers, respectively, was an acceptable primary goal of treatment among the treating physicians and patient's kin alike. Thus, very rarely etiological diagnosis was pursued. Besides, a lack of awareness among primary-care physicians regarding the various non-motor paretic manifestations of MMA coupled with extreme unavailability of appropriate radiological diagnostic tools, especially in the rural and suburban areas in India, further contributes to diagnostic delays. However, misdiagnosis is not rare in the developed Western countries as well, where mean diagnostic latencies of 5.3 years were observed. However, comparisons must be made with caution due to intrinsic differences in health policies between the two countries. Furthermore, due to various religious and cultural misbeliefs with a prevalent ignorance regarding self-health issues, a questionable appreciation, and attribution of initial symptoms among our patients must be raised (14, 56).

The commonest disease presentation in our patients was ischemic cerebral symptoms, conforming to most previous reports (4, 7, 10, 14, 15, 17, 18, 21, 36, 57). Cerebral infarction and TIA comprised 62.3 and 72.0% of the disease presentation among adults and children, respectively. Hemorrhagic MMA was noted only among adults. However, only 17.6% of adult patients had a hemorrhagic presentation. This lower incidence of hemorrhage is similar to the observations from recent studies among Western countries, and in stark contrast to the high rates of hemorrhagic MMA seen in the Japanese and Korean populations. It is hypothesized that a slower rate of progression of occlusive vasculopathy, allowing more time for the development of appropriate collaterals coupled with pathological differences in the fragility of collaterals might be responsible for less hemorrhage in patients of the USA in comparison to Japan. Extrapolation of the same in our population may not be far-fetched. Recent evidence suggests that choroidal collaterals carry a high risk for the recurrence of hemorrhage in MMA. Due to limited resources, only 20% of patients in our study underwent DSA, thus preventing comparison. Thus, future angiographic studies evaluating the characteristics of angioarchitecture in Indian MMA patients are warranted (7, 10, 13, 14, 36, 58, 59).

Both hemodynamic and thrombotic mechanisms can lead to infarcts in MMA. MMA leads to several characteristic infarct patterns, uncommon in conventional stroke, often not fitting with the classical vascular territory. Additionally, age-related differences exist in the frequency of these infarct patterns in MMA, conforming to dynamic features of arteriopathy, changes in collateral networks through the life span, and distortion of classical vascular territories (4, 14, 60). In accordance with the previous studies, we observed that the commonest region of insult was the cerebral cortex (45%) in our population (14, 60). The gyral pattern was significantly higher among children (*p* = 0.004) and corroborates with the selective vulnerability of cortical gray matter in children due to its high metabolic demand leading to severe damage on minor ischemic insults. Watershed or borderzone was found to occur in higher frequency (40%) in our cohort compared to the Korean population (5.5%) (60). However, a similar observation was made in a previous Indian study (24). This discrepancy may be speculated due to differences in the development of anastomotic connections between basal moyamoya vessels and terminal medullary branches in our population. Bleed in MMA occurs secondarily to rupture of friable collaterals harboring microaneurysm. Less commonly it may be due to rupture of associated true aneurysms around the circle of Willis. Similar to infarct patterns, atypical intracerebral sites and intraventricular regions are commonly involved. Our study showed the occurrence of primary intraventricular hemorrhage (IVH) in 33.3% of cases, in line with previous studies. Periventricular cortical microbleeds and dilated anterior choroidal artery branches serve as surrogate imaging markers for future risk of IVH. Thus, MMA should be considered in any young patient presenting with hemorrhage in atypical cerebral sites, without any traditional vascular risk factor (4, 14).

A substantial number of patients in our cohort (65%) had evidence of cerebral atrophy on brain imaging at the time of diagnosis. The diagnostic latency was significantly higher among patients with brain atrophy in comparison to those without (*p* < 0.001). An advanced Suzuki staging was observed (mean ± S.D, 3.58 ± 1.037) and it positively correlated to the diagnostic latency. “Ivy” sign, considered as an indirect radiological marker of decreased cerebrovascular reserve (57), was seen in 71.9% of our patients at the time of diagnosis. All this points to the fact that the brain was subjected to a longer duration of hypoperfusion and advanced disease presentation in our population, possibly an effect of delayed diagnosis, thus portending a poor prognosis.

A high frequency of posterior circulation involvement (55.6%) on angiography was seen in our population, nearly twice as high as the observed values among Asian countries and six times higher as compared to Western countries (14, 23, 61–63). Few previous studies from India had similar observations of high rates of posterior circulation involvement (14, 27, 64). However, posterior circulation stroke was observed in only 6.3% of the patients in our cohort keeping in line with its proposed rarity. Posterior cerebral artery (PCA) involvement is considered a natural disease process in MMA. The severity of steno-occlusive lesions of PCA strongly correlates with cerebral ischemia, probably due to worsening of collateral supply from PCA in the maintenance of anterior circulation. It indicates a rapid progression of the disease and is thus considered a poor prognostic marker in MMA. Recent studies have shown an early onset and aggressive PCA involvement in MMA patients with homozygous c.14576G > A variant of RNF 213. Future targeted genetic studies in the Indian population might shed some light on this deviant behavior (4, 14, 65).

## Conclusion

The epidemiology of MMA differs in various ways, inter-continental and intra-continental discrepancies are evident, corroborated by the subtle aberrations in the epidemiology of Indian MMA compared to other Asian countries like Japan and Korea. MMA contributes significantly to the etiological consideration of childhood stroke and must be considered during its evaluation. Though sex predilection was not very pronounced in our cohort, the presence of bimodal peak of age distribution, similar to other regions, hints toward a common underlying pathophysiological basis. Transient neurological symptoms are common, often subtle, and are frequently overlooked, leading to delayed diagnosis, especially true for countries with limited resources. Brain imaging is frequently characterized by the presence of stigma of hypoperfusion either in form of global or focal atrophy and the presence of “ivy” sign. Mechanisms of stroke underpinning MMA in the majority were appreciated to be as hemodynamic and vaso-occlusive for ischemic stroke and rupture immature, fragile collaterals in case of hemorrhagic presentation. A higher frequency of posterior circulation involvement was striking in our cohort. For appreciation, analysis, and a better understanding of these differences in the epidemiological profile of MMA, a nationwide registry is mandated.

## Data Availability Statement

The original contributions presented in the study are included in the article/supplementary material, further inquiries can be directed to the corresponding author.

## Ethics Statement

The studies involving human participants were reviewed and approved by Institute of Post Graduate Medical Education & Research, Kolkata, India. Written informed consent to participate in this study was provided by the participants' legal guardian/next of kin.

## Author Contributions

ShD: conceptualization, data curation, formal analysis, investigation, methodology, resources, supervision, visualization, statistical analysis, writing—original draft, and writing—review and editing. SoD: conceptualization, formal analysis, investigation, methodology, resources, supervision, visualization, and writing—review and editing. SuD: writing-review and editing. AH: methodology and statistical analysis. AP: conceptualization, supervision, visualization, and writing—review and editing. RG: data curation and writing-review and editing. BR: conceptualization, formal analysis, investigation, methodology, resources, project administration, supervision, visualization, and writing-review and editing. All the authors agreed upon the final form of the manuscript before submission. All authors contributed to the article and approved the submitted version.

## Conflict of Interest

The authors declare that the research was conducted in the absence of any commercial or financial relationships that could be construed as a potential conflict of interest.

## Publisher's Note

All claims expressed in this article are solely those of the authors and do not necessarily represent those of their affiliated organizations, or those of the publisher, the editors and the reviewers. Any product that may be evaluated in this article, or claim that may be made by its manufacturer, is not guaranteed or endorsed by the publisher.
